# Favorable Adherence and Tolerability of Letermovir Versus Valganciclovir for Cytomegalovirus Prophylaxis in Kidney Transplant Recipients Aged 65 y or Older: Findings From a Randomized, Phase 3 Trial

**DOI:** 10.1097/TXD.0000000000001990

**Published:** 2026-08-21

**Authors:** Suphamai Bunnapradist, Flavio Vincenti, Oliver Witzke, Stefan Schneeberger, M. Hong Nguyen, Natalya Broyde, Christopher L. Gilbert, Barbara Haber

**Affiliations:** 1 Division of Kidney Transplantation, David Geffen School of Medicine, University of California, Los Angeles, CA.; 2 Division of Nephrology, University of California, San Francisco School of Medicine, San Francisco, CA.; 3 Department of Infectious Diseases and Nephrology, West German Centre of Infectious Diseases and Immunology, University Medicine Essen, University Duisburg-Essen, Essen, Germany.; 4 Department of General, Transplant and Thoracic Surgery, Medical University of Innsbruck, Innsbruck, Austria.; 5 Department of Medicine, University of Pittsburgh, Pittsburgh, PA.; 6 Merck & Co., Inc., Rahway, NJ.

## Abstract

**Background.:**

Older patients undergoing kidney transplantation are at risk of perioperative complications, underscoring the need for optimized care. The number of kidney transplants in older adults has increased in the past decade, with greater reliance on transplants from deceased donors.

**Methods.:**

The phase 3, randomized, double-blind, active-controlled P002 study evaluated 200 d of letermovir versus valganciclovir prophylaxis in cytomegalovirus-seronegative kidney transplant recipients who received kidneys from cytomegalovirus-seropositive donors. Outcomes among participants aged 65 y or older are reported.

**Results.:**

Forty-eight and 55 participants aged 65 y or older received letermovir or valganciclovir, respectively. A lower percentage of participants receiving letermovir versus valganciclovir had committee-confirmed cytomegalovirus disease through week 52 posttransplant (9% versus 22%; stratum-adjusted difference, –12% [95% confidence interval [CI], –27% to 2%]), without significant differences in cytomegalovirus disease or viremia between groups. A significantly higher percentage of participants receiving letermovir had ≥90% prophylaxis adherence (96% [95% CI, 86%-100%] versus 49% [95% CI, 35%-63%]). The letermovir group showed numerically lower incidences of drug-related adverse events (25% versus 49%) and study medication discontinuations due to adverse events (10% versus 20%). A significantly lower percentage of participants receiving letermovir had leukopenia or neutropenia events through week 28 posttransplant (31% [95% CI, 19%-46%] versus 62% [95% CI, 48%-75%]). Participants receiving letermovir had numerically lower rates of opportunistic infections (11% versus 25%) and rehospitalizations (51% versus 69%) through week 52 posttransplant.

**Conclusions.:**

Letermovir showed substantial advantages over valganciclovir in adherence, safety, and tolerability, supporting its use as an important option for cytomegalovirus disease prophylaxis for older patients undergoing kidney transplantation.

**Clinical trial registration number.:**

ClinicalTrials.gov, NCT03443869

## Graphical abstract

**Figure:**
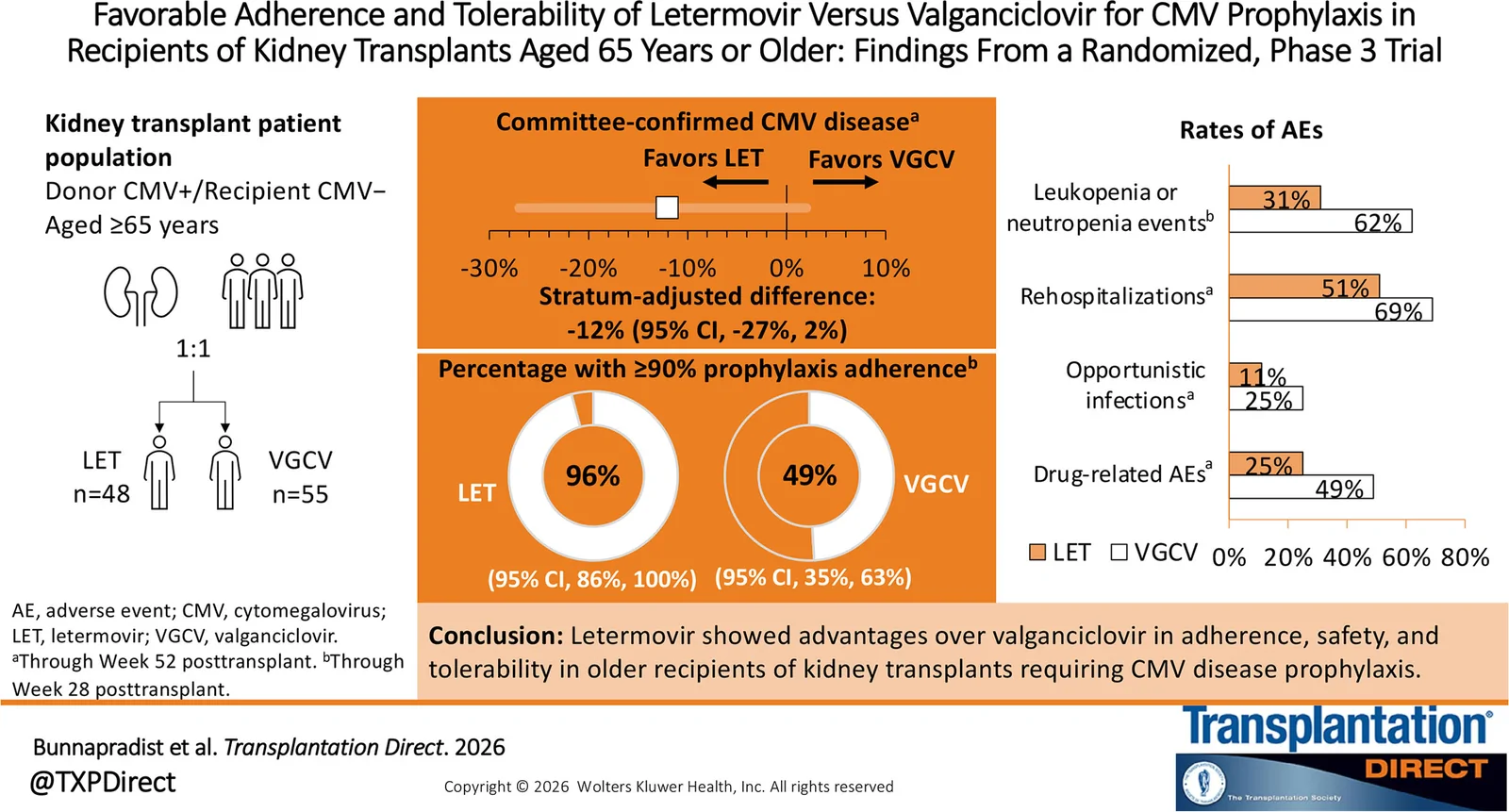

## INTRODUCTION

Older adults represent a growing population in need of kidney transplants, comprising 20% of candidates on the US kidney transplant waiting list in 2011, increasing to 26% by 2024.^1,2^ This shift brings clinical challenges. Older patients receiving kidney transplants are at greater risk of perioperative complications than younger patients.^3,4^ Immunosenescence, increased susceptibility to opportunistic infections (OIs), and infection-related mortality are pronounced in older adults, with infections being a major cause of death among older patients receiving kidney transplants.^3-7^ Older patients also have a higher likelihood of experiencing multiple comorbidities, frailty, and cognitive impairment,^4^ as well as longer kidney transplant wait times and an increased reliance on kidney transplants from deceased donors,^2,8,9^ all of which put them at risk of poor outcomes. Optimized clinical care is vital to address complex needs in this important population.

Cytomegalovirus (CMV) substantially contributes to morbidity and mortality in kidney transplant recipients.^6,10,11^ The risk of CMV disease is highest among those who are CMV seronegative and receive kidneys from donors who are CMV seropositive (CMV D^+^/R^−^).^6,10,11^ Higher rates of CMV viremia and reactivation have been found in older adults, supporting heightened susceptibility to CMV disease.^3,12^

CMV prophylaxis is associated with improved posttransplant outcomes, including lower rates of CMV disease, reduced mortality, fewer graft rejections, and improved graft function and survival.^13-15^ For >2 decades, valganciclovir was the only antiviral approved for CMV disease prophylaxis in kidney transplant recipients. However, valganciclovir has critical limitations, especially among older adults, and it must be used with caution in this population.^16^ It is eliminated via the kidneys, requiring dose adjustments based on creatinine clearance (CrCl), leading to many patients with impaired kidney function having complex, intermittent dosing schedules.^16-18^ Frequent dose adjustments, particularly in the immediate posttransplant period when donor kidney function is variable as it recovers, can burden both healthcare providers and patients, and are associated with higher rates of CMV DNAemia and nonadherence.^16-20^

Letermovir was approved in the United States in 2023 for CMV disease prophylaxis in adult kidney transplant recipients who were at high risk of acquiring CMV (ie, those who are CMV D^+^/R^−^).^21^ The approval was based on results from the pivotal phase 3, randomized, noninferiority P002 clinical trial, which demonstrated that letermovir is noninferior to valganciclovir for CMV disease prophylaxis in this population.^22^ Letermovir was associated with a higher rate of adherence and lower rates of neutropenia, leukopenia, prophylaxis discontinuation, and emergent CMV resistance compared with valganciclovir.^22,23^ Letermovir offers additional benefits over valganciclovir, including a lower risk of myelotoxicity, a simpler dosing regimen, and a unique mechanism of action that minimizes the potential for cross-resistance with other CMV therapies.^22^ Letermovir requires no renal dose adjustment because it is not eliminated via the kidneys.^21^ However, letermovir has a greater drug-drug interaction potential, although it is manageable.^21^ Letermovir also lacks activity against herpes simplex virus and varicella zoster virus, which are covered by valganciclovir.^22^

Given the importance of optimizing care for the growing population of older adults undergoing kidney transplantation, it is crucial to evaluate CMV disease prophylaxis options in this population. In this study, efficacy, adherence, safety and tolerability, and health outcomes associated with letermovir and valganciclovir in kidney transplant recipients aged 65 y or older who were CMV D^+^/R^−^ were evaluated.

## MATERIALS AND METHODS

### Design

This prespecified subanalysis focuses on participants aged 65 y or older in the phase 3, randomized, active-controlled, double-blind, double-dummy, noninferiority P002 clinical trial conducted across 94 participating sites in 16 countries (ClinicalTrials.gov identifier: NCT03443869). The trial was initiated in May 2018 and was impacted by the COVID-19 pandemic—the first participant visit occurred on May 3, 2018, and the last occurred on April 5, 2022. A detailed overview of the trial methods, including the protocol and statistical analysis plan, has been previously published.^22^

Participants were randomized (1:1) to receive letermovir or valganciclovir, each with a matching placebo, with stratification based on the receipt of lymphocyte-depleting induction immunosuppression (**Figure S1, SDC,** https://links.lww.com/TXD/A893). Prophylaxis started within 7 d after kidney transplantation and continued through week 28. Participants assigned to letermovir received oral letermovir 480 mg once daily (240 mg if receiving concomitant cyclosporine-A), oral acyclovir 400 mg twice daily (for prophylaxis against herpes simplex virus and varicella zoster virus), and a valganciclovir placebo. Those assigned to valganciclovir received oral valganciclovir 900 mg once daily and matching placebo for letermovir and acyclovir. Intravenous formulations of study drugs were provided, if necessary. Doses of valganciclovir and acyclovir were titrated for kidney function based on CrCl assessment at each study visit as previously described^18^; further details are provided in **Supplemental Methods** (**SDC,** https://links.lww.com/TXD/A893).

### Ethics

The trial was conducted in accordance with the standards of the International Conference on Harmonization Good Clinical Practice Guideline and all applicable laws, rules, and regulations relating to the conduct of clinical trials. The study protocol and amendments (final protocol, MK-8228-002-05) were approved by the appropriate institutional review boards and regulatory agencies at all sites. All participants provided written informed consent.

### Participants

Participants were adults who were CMV seronegative and received a kidney transplant from a donor who was CMV seropositive. A key exclusion criterion was posttransplant CrCl ≤10 mL/min at randomization. Race and ethnicity were self-reported by participants. Efficacy and health outcomes were analyzed in the full analysis set comprising participants who received ≥1 dose of study medication, were CMV D^+^/R^−^, and were negative for CMV DNAemia at baseline (day 1). Safety was assessed in all randomized participants who received ≥1 dose of study medication (all participants as treated [APaT] population).

Kidney function across prophylaxis groups in participants younger than 65 y and 65 y or older was assessed to determine whether posttransplant kidney function was more affected in the older age group.

### Endpoints

The primary efficacy endpoint was the incidence of adjudication committee-confirmed (henceforth referred to as “committee-confirmed”) CMV disease through week 52 posttransplant. A secondary efficacy endpoint was the incidence of committee-confirmed CMV disease through week 28 posttransplant. The incidence of quantifiable CMV DNAemia through week 52 posttransplant was an exploratory efficacy outcome. Evaluating the safety and tolerability of letermovir versus valganciclovir was a secondary endpoint. Safety and tolerability were assessed using accumulated safety data, including adverse events (AEs) and laboratory findings, categorized according to MedDRA version 25.0. Laboratory findings were evaluated according to predefined criteria, including defined limits and change from baseline. A prespecified key safety outcome was the percentage of participants with a composite outcome of neutropenia- or leukopenia-related events during prophylaxis (through week 28 posttransplant), defined as (1) an investigator-reported AE of leukopenia, (2) an investigator-reported AE of neutropenia, (3) white blood cell count <3500 cells/μL, or (4) absolute neutrophil count <1000 cells/μL. Additionally, neutropenia- or leukopenia-related events among participants aged 65 y or older were assessed by use of lymphocyte-depleting induction therapy post hoc. Health outcomes through weeks 28 and 52 posttransplant were the percentage of participants with allograft dysfunction and/or rejection (including kidney function assessed through estimated glomerular filtration rate [eGFR]), rehospitalizations, and select OIs.

Kidney function assessed via CrCl in the letermovir and valganciclovir groups through week 28 posttransplant was analyzed post hoc. The association between dose frequency and adherence to the active CMV antiviral was also analyzed post hoc, with dose frequency characterized as the percentage of participants who received once daily dosing (no intermittent dosing) versus intermittent dosing (every 2 d or twice weekly) for letermovir and valganciclovir placebo (letermovir group) and valganciclovir and letermovir placebo (valganciclovir group).

### Procedures

Incidence of CMV disease, reported as “CMV end-organ disease” or “CMV syndrome,” was assessed by the investigator at screening; baseline; and weeks 1, 2, 4, 6, 8, 10, 12, and every 4 wk thereafter through week 52 posttransplant. Assessments were also conducted when CMV was suspected by the investigator. All investigator-reported cases of CMV disease were assessed by an external masked clinical adjudication committee using established diagnostic criteria from the Disease Definitions Working Group of the Cytomegalovirus Drug Development Forum.^24^ CMV DNAemia was assessed from plasma samples collected at baseline, weeks 2 and 4, and every 4 wk thereafter through week 52 posttransplant. Additional assessments were performed at the onset of suspected or confirmed CMV disease or initiation of CMV treatment. Quantification was performed using DNA–polymerase chain reaction by a central laboratory, as previously described.^22^ Quantifiable CMV DNAemia was defined as any detected CMV with a numeric value ≥137 IU/mL, with any level above this threshold regarded equally. Health outcomes, including rehospitalizations, select OIs, biopsy-proven acute renal graft rejections, and graft loss, were assessed at baseline and weeks 1, 2, 4, 6, 8, 10, 12, and every 4 wk thereafter through week 52 posttransplant.

A study medication discontinuation visit was initiated when an investigator confirmed CMV disease or discontinued prophylaxis to start CMV treatment. Participants who discontinued study medication before week 28 posttransplant completed all remaining study visits. Adherence to blinded study medication was self-reported by participants in a medication diary. Site personnel verified medication diary accuracy by comparing entries with the quantities of the returned study drugs. Adherence was quantified as the number of days a participant was on therapy divided by the number of days the participant should have been on therapy, multiplied by 100. Investigator-prescribed dose modifications based on CrCl were not counted as nonadherent days.

A metabolic panel was conducted on blood samples collected from participants at baseline, weeks 1 and 4, and every 4 wk thereafter through week 32 posttransplant. Dose adjustments for valganciclovir (or intravenous ganciclovir) and acyclovir were based on CrCl calculated using the Cockcroft-Gault equation, in accordance with prescribing information and prophylaxis guidelines.^16,25-27^ The 4-variable Modification of Diet in Renal Disease equation was used to calculate eGFR.^28^

### Statistics

Across statistical analyses, due to the expected smaller sample size in the subpopulation, wider 95% confidence intervals [CIs] were expected, and *P* values were not calculated. Nonoverlapping 95% CIs between prophylaxis groups were considered statistically significant. The estimate of the effect of letermovir compared with valganciclovir was evaluated for the percentage of participants with committee-confirmed CMV disease through week 52 posttransplant, calculated using the stratum-adjusted Mantel-Haenszel method,^29^ with stratification by receipt of lymphocyte-depleting induction immunosuppression. The observed failure approach was used in this analysis, which excluded participants who discontinued the study early for any reason. The percentage of participants with quantifiable CMV DNAemia through weeks 28 and 52 posttransplant was evaluated similarly. Differences (and corresponding 95% CIs) in the composite safety outcome of leukopenia and neutropenia through week 28 posttransplant, as well as for adherence, rehospitalization, and allograft dysfunction and/or rejection, were estimated using the Miettinen-Nurminen method.^30^ Statistical tests for adherence, rehospitalizations, and allograft dysfunction and/or rejection were performed post hoc. Descriptive statistics were used to analyze CrCl through week 28 posttransplant, CMV antiviral dosing frequency and adherence, and serious AEs (SAEs) leading to rehospitalizations.

## RESULTS

### Participant Disposition

In the APaT population, participants aged 65 y or older comprised 16% (48/292) of those in the letermovir group and 19% (55/297) in the valganciclovir group (**Figure S2, SDC,** https://links.lww.com/TXD/A893). One participant in the letermovir group was excluded from the full analysis set due to having CMV DNAemia at baseline; none were excluded in the valganciclovir group. Through week 52 posttransplant, a lower percentage of participants aged 65 y or older discontinued study medication in the letermovir versus valganciclovir group (25% [12/48] versus 40% [22/55]).

### Baseline Characteristics

Most participants were of White race, and approximately two-thirds were men (**Table S1, SDC,** https://links.lww.com/TXD/A893). Demographic and baseline characteristics were generally well matched between prophylaxis groups. A high percentage of participants in both groups received kidneys from a deceased donor (letermovir, 73%; valganciclovir, 84%). A slightly lower percentage of participants in the letermovir group versus the valganciclovir group received lymphocyte-depleting induction immunosuppression (letermovir, 38%; valganciclovir, 47%), of which antithymocyte globulin was the predominant lymphocyte-depleting immunosuppression agent. Compared with participants younger than 65 y, those aged 65 y or older were more likely to have a kidney transplant from a deceased donor (**Table S2, SDC,** https://links.lww.com/TXD/A893).

Use of concomitant immunosuppressants during prophylaxis is presented in **Table S3** (**SDC,** https://links.lww.com/TXD/A893). The most frequently reported (>50%) were tacrolimus, mycophenolate mofetil, and prednisone, with generally comparable rates across prophylaxis groups.

### Committee-confirmed CMV Disease

**FIGURE 1.**
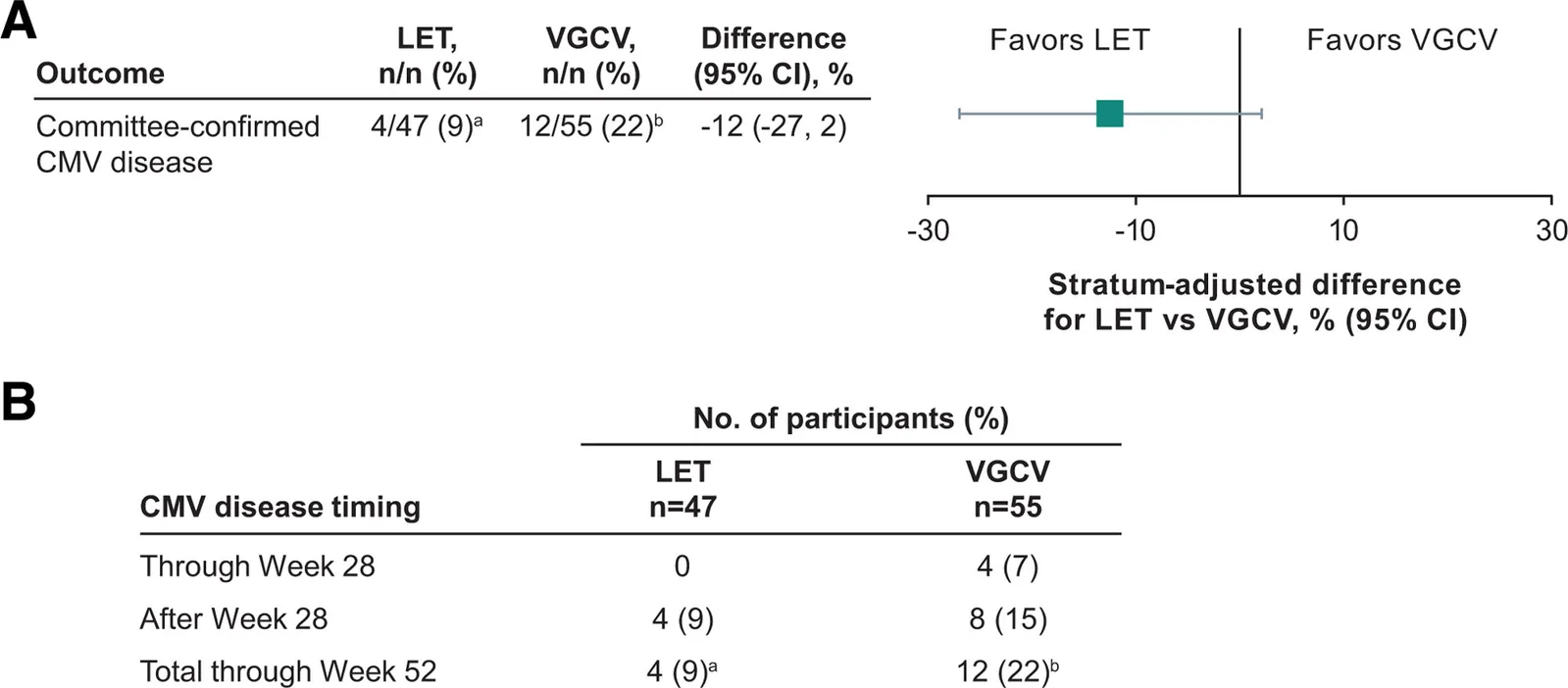
Committee-confirmed CMV disease (FAS). A, Percentage of participants with committee-confirmed CMV disease through week 52 posttransplant and stratum-adjusted difference. B, Percentage of participants who developed committee-confirmed CMV disease during prophylaxis (through week 28 posttransplant) and after prophylaxis cessation (after week 28 posttransplant). CI, confidence interval; CMV, cytomegalovirus; FAS, full analysis set; LET, letermovir; VGCV, valganciclovir. ^*a*^All 4 participants had CMV syndrome. ^*b*^One participant had CMV end-organ disease and 11 participants had CMV syndrome.

Through week 52 posttransplant, 4 of 47 and 12 of 55 participants in the letermovir and valganciclovir groups, respectively, had committee-confirmed CMV disease (9% [95% CI, 2%-20%] versus 22% [95% CI, 12%-35%]; stratum-adjusted difference, –12% [95% CI, –27% to 2%]; Figure 1). All 4 participants in the letermovir group had CMV syndrome; 11 and 1 participants in the valganciclovir group had CMV syndrome or CMV end-organ disease, respectively.

During 28 wk of posttransplant prophylaxis, no participants in the letermovir group developed CMV disease, compared with 7% (4/55) in the valganciclovir group. Of these 4 participants in the valganciclovir group with CMV disease during prophylaxis, 2 had breakthrough viremia, 1 had viremia on the last day of prophylaxis that was later diagnosed as CMV disease, and 1 had CMV disease after valganciclovir premature discontinuation due to leukopenia.

### Quantifiable CMV DNAemia

Through week 28 posttransplant, the incidence of quantifiable CMV DNAemia was nonsignificantly lower for participants in the letermovir versus valganciclovir group (2% [95% CI, 0%-11%] versus 15% [95% CI, 6%-27%]; **Figure S3, SDC,** https://links.lww.com/TXD/A893). Through week 52 posttransplant, the incidence of quantifiable CMV DNAemia was slightly and numerically lower in the letermovir versus valganciclovir group (32% [95% CI, 19%-47%] versus 38% [95% CI, 25%-52%]).

### Adherence and Dosing

**FIGURE 2.**
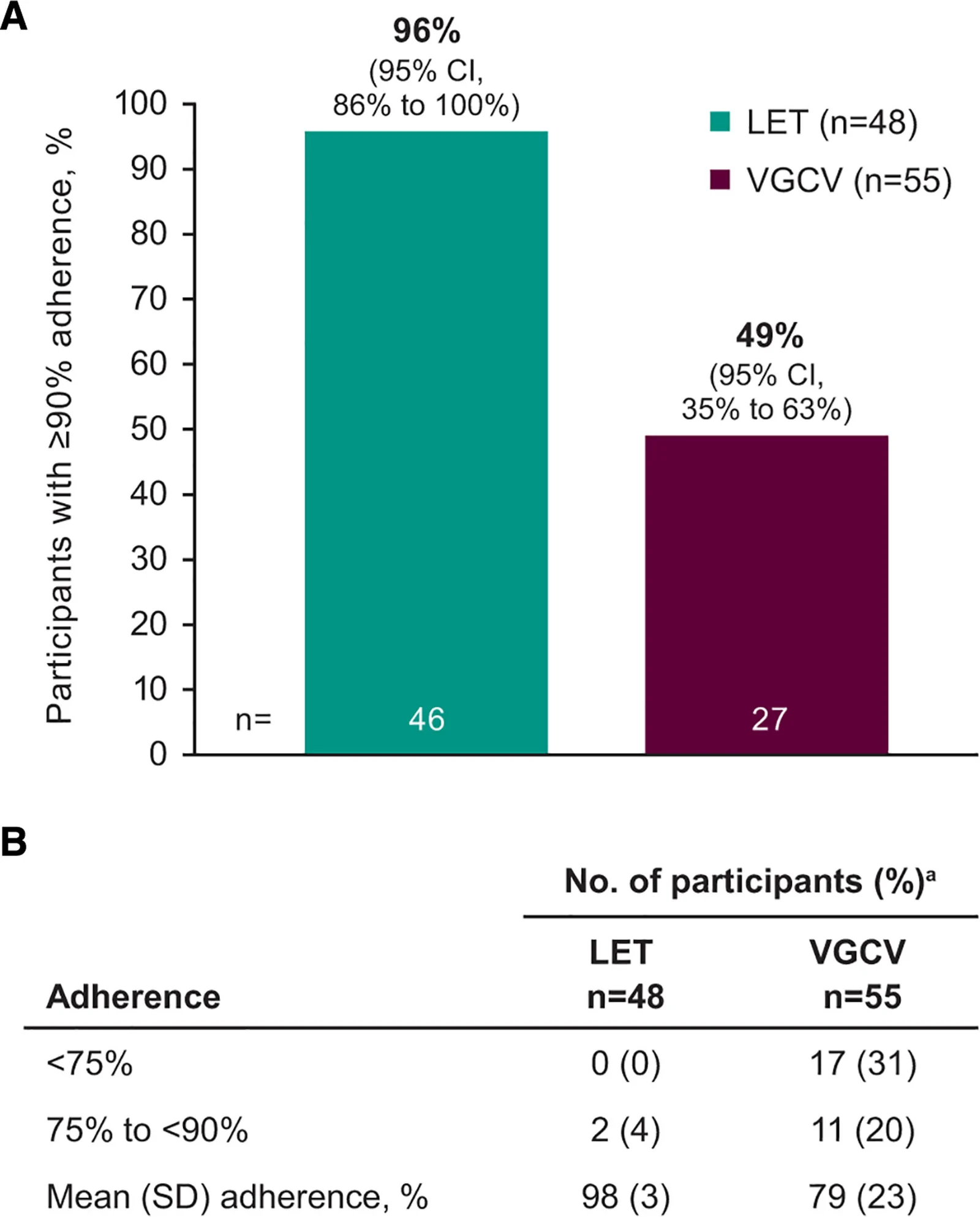
Adherence to active anti-CMV study medication through week 28 posttransplant (APaT). A, Percentage of participants with ≥90% adherence. B, Percentage of participants with <75% and 75% to <90% adherence and overall mean (SD) adherence rates. APaT, all participants as treated; CI, confidence interval; CMV, cytomegalovirus; LET, letermovir; VGCV, valganciclovir. ^*a*^Except for mean (SD) adherence, which is presented as the overall adherence rate.

A significantly higher percentage of participants had ≥90% adherence through week 28 posttransplant in the letermovir versus valganciclovir group (96% [95% CI, 86%-100%] versus 49% [95% CI, 35%-63%]; Figure 2A). No participants in the letermovir group had an adherence rate <75%, compared with 31% (17/55) of participants in the valganciclovir group (Figure 2B). Overall adherence mean (SD) was 98% (3%) and 79% (23%) in the letermovir and valganciclovir groups, respectively.

All participants were prescribed once daily letermovir or letermovir placebo (**Figure S4, SDC,** https://links.lww.com/TXD/A893). In the valganciclovir group, 24% were prescribed once daily valganciclovir (450 or 900 mg), and the remaining 76% were prescribed intermittent valganciclovir (450 mg every 2 d or twice weekly) during all or part of prophylaxis.

In the letermovir group, where 100% of participants received once daily letermovir, most participants had 100% or 90% to <100% adherence (54% [26/48] and 42% [20/48], respectively; **Figure S4, SDC,** https://links.lww.com/TXD/A893). The percentages of participants with 100% or 90% to <100% adherence were comparable in participants prescribed once daily valganciclovir (31% [4/13] and 62% [8/13], respectively). Adherence was worse among participants in the valganciclovir group prescribed intermittent dosing. No participants achieved 100% adherence; 36% (15/42) had 90% to <100% adherence, and most had 75% to <90% or <75% adherence (26% [11/42] and 38% [16/42], respectively). Median adherence was 100%, 100%, and 82% for participants prescribed once daily letermovir, once daily valganciclovir, and intermittent valganciclovir, respectively.

### Posttransplant Kidney Function

Through week 28 posttransplant, participants aged 65 y or older had lower CrCl than those younger than 65 y (**Figure S5, SDC,** https://links.lww.com/TXD/A893). Among participants aged 65 y or older, mean (SE) CrCl was similar between prophylaxis groups at day 1 (letermovir, 35.1 [2.9] mL/min; valganciclovir, 33.1 [2.5] mL/min) and week 28 (letermovir, 50.8 [2.9] mL/min; valganciclovir, 50.5 [2.5] mL/min). Kidney function assessed using eGFR was consistent with the CrCl analysis (**Figure S6, SDC,** https://links.lww.com/TXD/A893).

#### Safety

**FIGURE 3.**
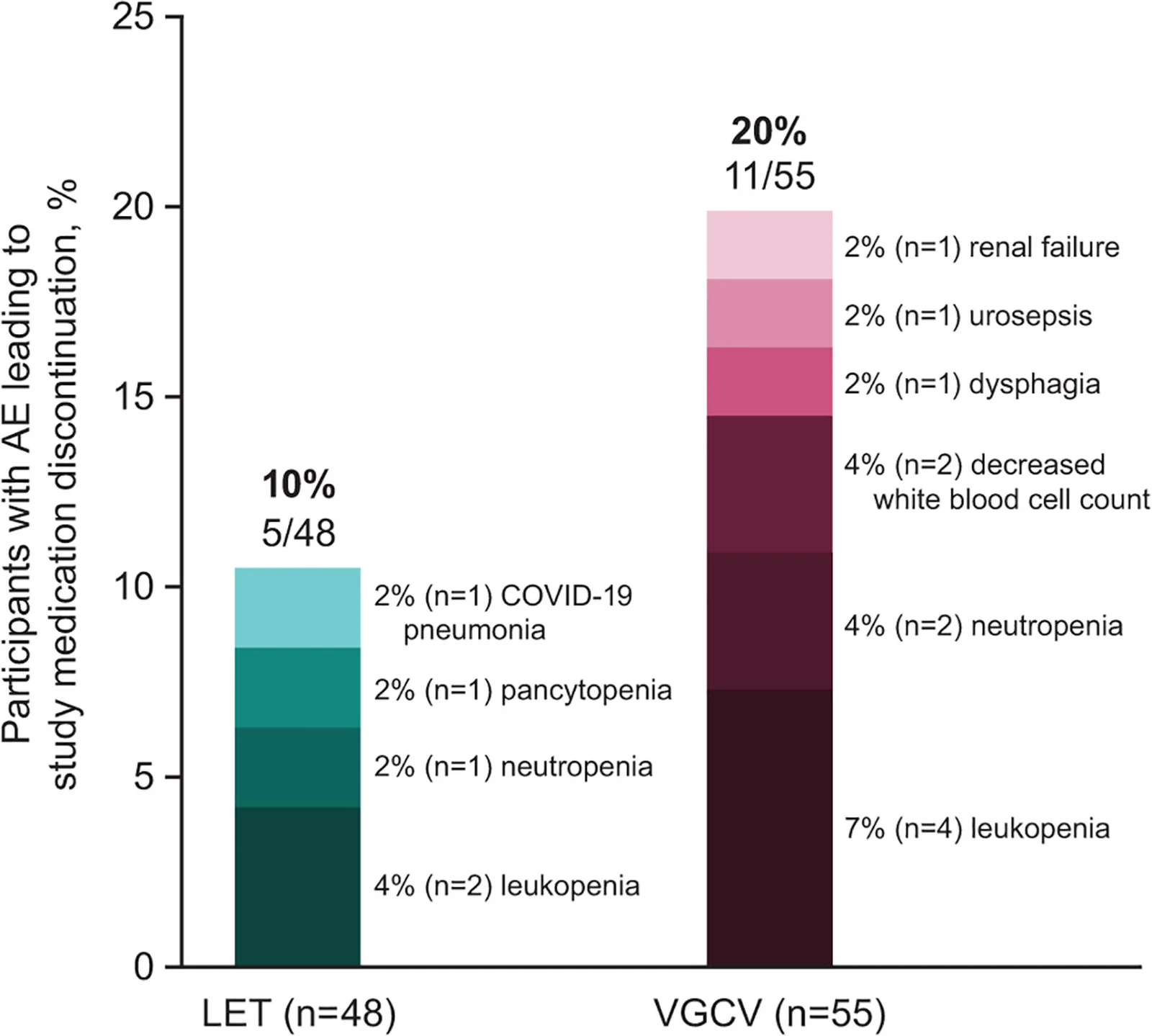
Adverse events leading to discontinuation of study medication (APaT). AE, adverse event; APaT, all participants as treated; LET, letermovir; VGCV, valganciclovir.

Most participants in both prophylaxis groups had ≥1 AE (Table 1). In the letermovir versus valganciclovir group, there were numerically lower incidences of drug-related AEs (25% versus 49%) and discontinuations of study medication due to drug-related AEs (8% versus 16%). There were no deaths in either group. A numerically lower percentage of participants had AEs leading to discontinuation of study medication in the letermovir versus valganciclovir group (10% versus 20%; Figure 3).

**TABLE 1.** Adverse events in participants aged 65 y or older (APaT)

	No. of participants (%)	
	LET (N = 48)	VGCV (N = 55)
AE summary		
Any AE	46 (96)	50 (91)
Drug-related AE^*a*^	12 (25)	27 (49)
Drug-related SAE^*a*^	0	3 (5)
SAE	20 (42)	25 (46)
Discontinued study medication due to an AE	5 (10)	11 (20)
Discontinued due to SAE	1 (2)	4 (7)
Discontinued due to drug-related AE^*a*^	4 (8)	9 (16)
Discontinued due to drug-related SAE^*a*^	0	2 (4)^*b*^
Death	0	0
Any AE during prophylaxis phase with ≥10% incidence		
Diarrhea	13 (27)	23 (42)
Tremor	13 (27)	15 (27)
Peripheral edema	13 (27)	15 (27)
Leukopenia	5 (10)	20 (36)
Constipation	5 (10)	11 (20)
Urinary tract infection	9 (19)	10 (18)
Hypomagnesemia	5 (10)	10 (18)
Hypophosphatemia	7 (15)	7 (13)
Hypertension	7 (15)	6 (11)
Hyperkalemia	7 (15)	6 (11)
Hyperglycemia	5 (10)	6 (11)
Acute kidney injury	5 (10)	6 (11)
Nausea	7 (15)	4 (7)
Hypotension	5 (10)	2 (4)
Anemia	4 (8)	10 (18)
Fatigue	3 (6)	10 (18)
Neutropenia	3 (6)	8 (15)
Increased blood creatinine	2 (4)	6 (11)
Decreased white blood cell count	0	6 (11)

^*a*^As determined by the investigator.

^*b*^Leukopenia and decreased white blood cell count in 1 participant each.

AE, adverse event; APaT, all participants as treated; LET, letermovir; SAE, serious AE; VGCV, valganciclovir.

#### Leukopenia or Neutropenia

**FIGURE 4.**
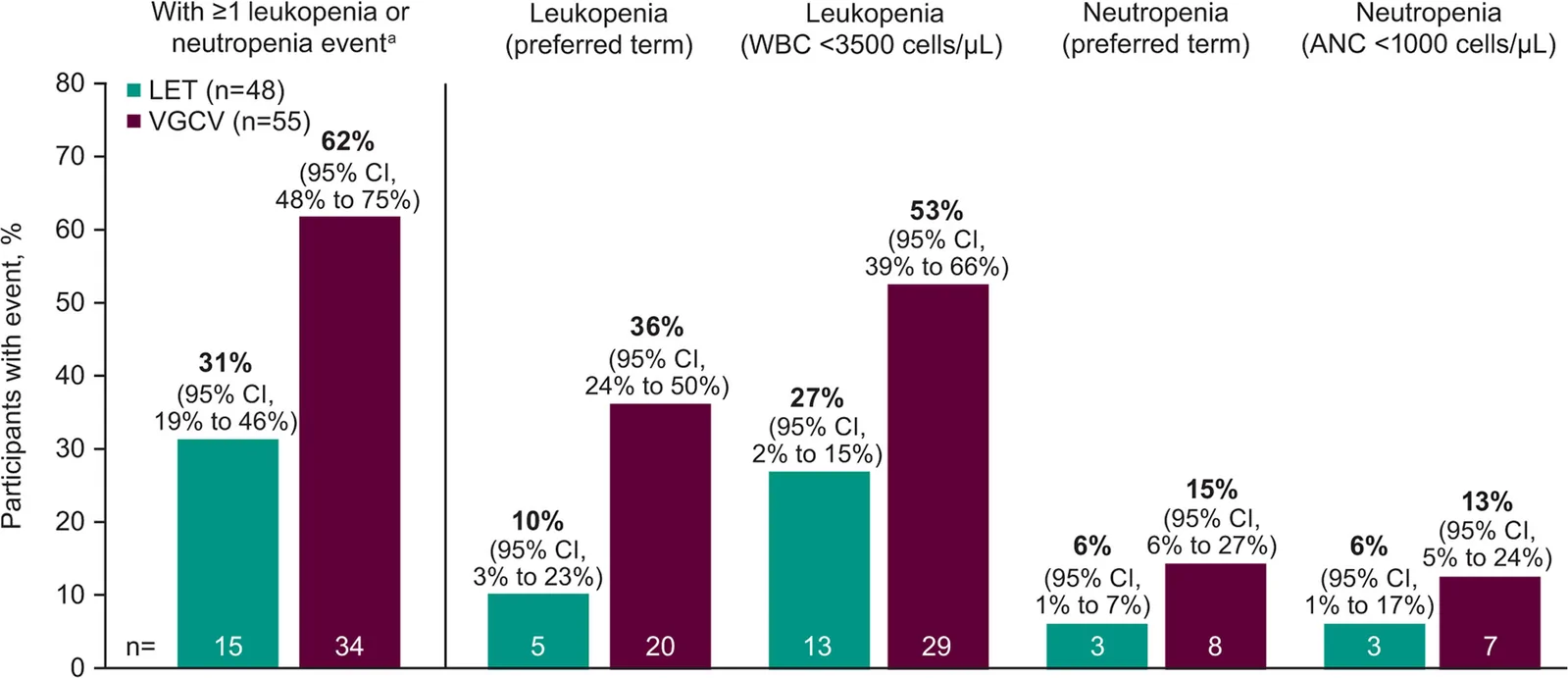
Composite safety endpoint of leukopenia or neutropenia events through week 28 posttransplant (APaT). Percentage of participants experiencing leukopenia or neutropenia and individual rates of each event. AE, adverse event; ANC, absolute neutrophil count; APaT, all participants as treated; CI, confidence interval; LET, letermovir; VGCV, valganciclovir; WBC, white blood cell. ^*a*^Prespecified composite safety endpoint tested for significance included any of the following during therapy: AE of leukopenia, total WBC count <3500 cells/μL, AE of neutropenia, or ANC <1000 cells/μL.

Through week 28 posttransplant, a significantly lower percentage of participants met criteria for the composite safety endpoint of leukopenia or neutropenia in the letermovir versus valganciclovir group (31% [95% CI, 19%-46%] versus 62% [95% CI, 48%-75%]; Figure 4). Across leukopenia or neutropenia event types, lower rates were consistently observed with letermovir versus the valganciclovir group (Figure 4). No meaningful differences in the incidences of leukopenia or neutropenia were observed by the use of lymphocyte-depleting induction immunosuppression (**Table S4, SDC,** https://links.lww.com/TXD/A893).

#### Select OIs

**FIGURE 5.**
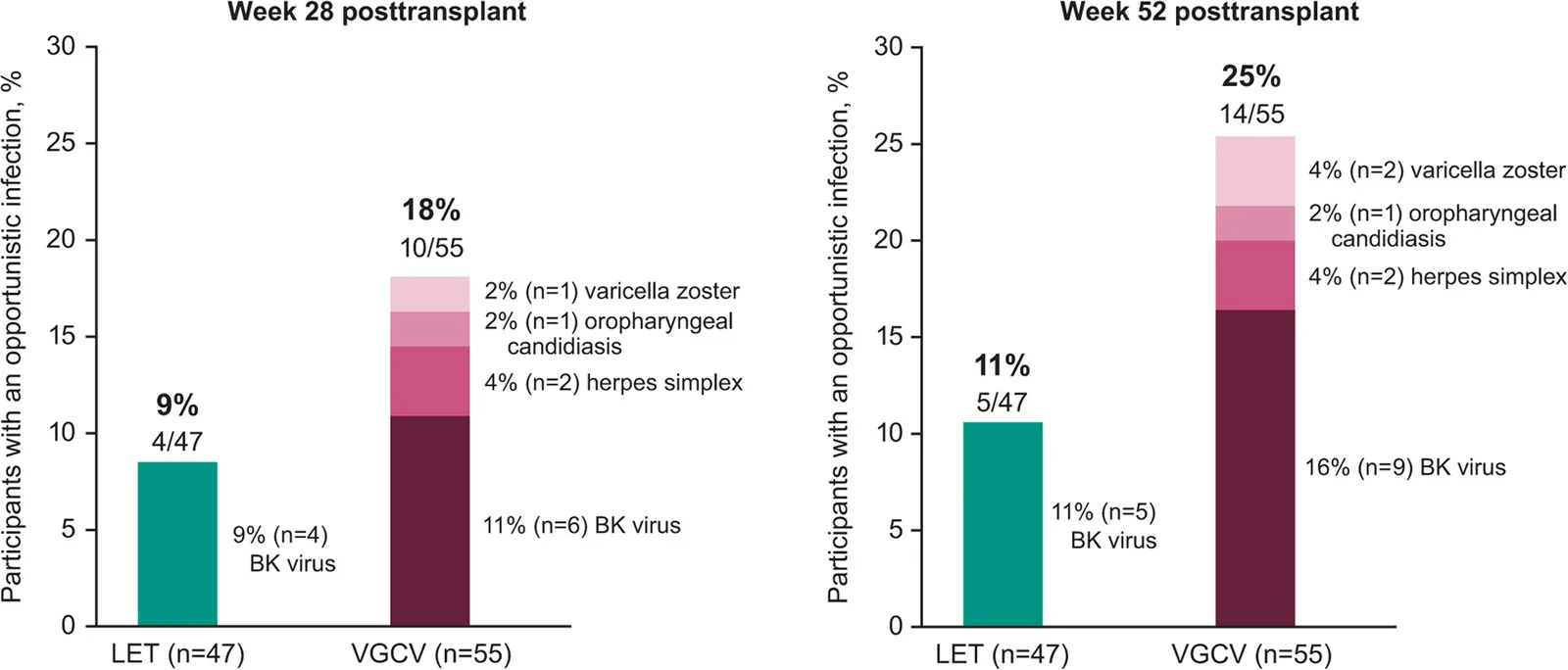
Percentage of participants with select opportunistic infections through weeks 28 and 52 posttransplant (FAS). FAS, full analysis set; LET, letermovir; VGCV, valganciclovir.

A numerically lower percentage of participants in the letermovir versus the valganciclovir group experienced OIs (week 28, 9% versus 18%; week 52, 11% versus 25%; Figure 5). BK virus was the most common opportunistic infection in both prophylaxis groups. Whereas BK virus was the only OI in the letermovir group, participants treated with valganciclovir also experienced herpes simplex virus, oropharyngeal candidiasis, and varicella zoster virus.

#### Rehospitalizations

Rehospitalization was common among older kidney transplant recipients in this study (**Figure S7, SDC,** https://links.lww.com/TXD/A893). A numerically lower percentage of participants in the letermovir versus the valganciclovir group experienced rehospitalizations through week 52 posttransplant (letermovir, 51% [95% CI, 36%-66%]; valganciclovir, 69% [95% CI, 55%-81%]). A similar trend was observed for participants with rehospitalizations due to CMV disease or infection (letermovir, 9% [95% CI, 2%-20%]; valganciclovir, 18% [95% CI, 9%-31%]). The mean duration of rehospitalizations was 12.4 d (range, 1–57) in the letermovir group and 18.8 d (range, 1–142) in the valganciclovir group. For rehospitalizations due to CMV disease, the mean duration was 9.3 d (range, 2–17) in the letermovir group and 17.0 d (range, 2–60) in the valganciclovir group.

Among the SAEs associated with rehospitalization, acute kidney injury was the most common event, occurring in none of the participants in the letermovir group and 11% (4/38) of participants in the valganciclovir group (**Table S5, SDC,** https://links.lww.com/TXD/A893).

#### Allograft Dysfunction

The percentage of participants experiencing allograft dysfunction and/or rejection was high and comparable between prophylaxis groups (**Table S6, SDC,** https://links.lww.com/TXD/A893). Through week 52 posttransplant, only 1 participant (in the valganciclovir group) had graft loss. A numerically higher percentage of participants had biopsy-proven acute renal graft rejection in the letermovir versus the valganciclovir group through week 28 and week 52 posttransplant; however, these results may have been affected by the COVID-19 pandemic, which reduced the number of biopsies conducted.

## DISCUSSION

Advantages of letermovir over valganciclovir for CMV prophylaxis in older participants were shown in the randomized phase 3 P002 trial. Letermovir prophylaxis was associated with nonsignificantly lower rates of committee-confirmed CMV disease and quantifiable CMV DNAemia through week 52 posttransplant. Letermovir demonstrated significantly better adherence over valganciclovir and favorable safety outcomes, with significantly lower rates of leukopenia or neutropenia events, and numerically lower rates of drug-related AEs and discontinuations. Participants receiving letermovir also had numerically lower rates of OIs and rehospitalizations. Together, these findings support the use of letermovir for optimized care in this population, providing simpler dosing and better outcomes compared with valganciclovir.

Posttransplant kidney function, evaluated by CrCl and eGFR, was more compromised in participants aged 65 y or older versus younger than 65 y, which was most likely due to the higher percentage of deceased donor kidneys (79% versus 56%). Additionally, lower kidney quality may be a factor in this older subpopulation, as studies have found that the probability of receiving lower-quality organs increases with age.^31,32^ Valganciclovir dosing requires adjustment based on CrCl,^16^ and frequent dose modifications have been associated with higher quantifiable CMV DNAemia and nonadherence rates.^18^ Consequently, the greater degree of compromised kidney function in the older subpopulation may necessitate more frequent dose adjustments, potentially impacting prophylaxis adherence and contributing to the risk of CMV DNAemia. Letermovir is not eliminated by the kidneys,^21^ enabling simpler dosing and higher adherence rates, thus helping manage the complex needs of older adults who are CMV D^+^/R^−^.

All participants in the letermovir group were prescribed once daily dosing. In contrast, 76% of the valganciclovir group received intermittent dosing. Adherence was high among participants on once daily prophylaxis but was moderate to poor for those prescribed intermittent valganciclovir. Valganciclovir prophylaxis in this population may impose a burden on patients and healthcare providers stemming from the need for more frequent clinic visits and difficulties in maintaining adherence.^33^ Disruptive medication schedules, twice daily versus once daily dosing, and changes to dosing are barriers to adherence in the kidney transplant setting.^34-36^

Letermovir showed a favorable safety and tolerability profile in older adults compared with valganciclovir—with numerically lower rates of drug-related AEs and discontinuations—and significantly lower rates of leukopenia or neutropenia events. The significantly reduced incidence of myelotoxicity-related events is especially beneficial to older adults who are at risk of immunosenescence and have elevated susceptibility to infections after kidney transplantation.^6^

A numerically higher rate of rehospitalizations was observed in the valganciclovir versus the letermovir group at week 52 posttransplant. This may be attributable to the higher incidence of CMV disease and other OIs, which were numerically higher in the valganciclovir group. Consistent with this trend, infections have been reported to be the number one risk factor for hospital readmission 1 y after transplantation in older adults.^3^ Herpes simplex and varicella zoster virus are sensitive to valganciclovir and ganciclovir.^37^ Surprisingly, 2 participants receiving valganciclovir had breakthrough herpes simplex infections and 1 had a varicella zoster virus infection. These breakthrough infections may have been related to lower adherence in the valganciclovir group. No participants in the letermovir group, compared with 4 in the valganciclovir group, had SAEs of acute kidney injury leading to rehospitalization.

Because the participants were relatively healthy, there were no deaths in this study, and allograft loss was rare. Data from real-world settings are needed to understand how prophylaxis choice could affect posttransplant outcomes.

These findings build on the overall findings from the P002 study,^22^ revealing novel insights in the older adult subpopulation. A numerically lower incidence of committee-confirmed CMV disease through week 52 posttransplant in older adults receiving letermovir compared with valganciclovir (9% versus 22%) was observed, whereas rates were comparable between prophylaxis groups in the overall population (letermovir, 10%; valganciclovir, 12%). Adherence to letermovir was more favorable than to valganciclovir, and this difference was more pronounced among older adults than in the overall population. Additionally, OIs—an important outcome in this high-risk population^5^—were numerically lower among participants aged 65 y or older in the letermovir group compared with the valganciclovir group (11% versus 25%) through week 52 posttransplant, whereas such rates were comparable between prophylaxis groups in the overall population (letermovir, 16%; valganciclovir, 17%).^38^ These findings support the advantages of letermovir for prophylaxis optimization in older adults.

Alongside its benefits, the use of letermovir warrants certain considerations. Healthcare providers should be aware of the drug-drug interaction profile of letermovir.^21^ Letermovir interacts with immunosuppressants, requiring monitoring of their concentrations and, in the case of cyclosporine, decreased letermovir dosage.^21^ Letermovir has a manageable drug-drug interaction profile, as described in the prescribing information.^21^ Although letermovir currently has a higher drug acquisition cost than valganciclovir, the introduction of generic formulations is expected to reduce costs.

### Strengths and Limitations

Strengths of this study include its focus on the population of adults aged 65 y or older, which is an important population at increased risk of perioperative complications and poor outcomes compared with younger recipients.^3,4^ As part of the robust, randomized, double-blind, and active-controlled P002 trial, results could be compared with both the overall study population and the younger (younger than 65 y) subpopulation. Additionally, an assessment of adherence to CMV prophylaxis showed comparable percentages of participants who were prescribed once daily letermovir (letermovir group) versus its matching placebo (valganciclovir group) and once daily or intermittent valganciclovir versus its matching placebo, validating that dosing was managed similarly in both groups.

Limitations of the overall analysis have been previously reported.^22^ Participants in the older subpopulation were nearly exclusively of White race. Testing for statistical significance was limited due to the expected smaller population size. Although demographic and baseline characteristics were generally well balanced across groups, the use of lymphocyte-depleting induction immunosuppression was 9% higher in the valganciclovir group; however, it had previously been demonstrated that the use of lymphocyte-depleting induction immunosuppression did not affect committee-confirmed CMV disease incidence between the letermovir and valganciclovir groups.^22^ Results on biopsy-proven acute renal graft rejection may have been affected by the COVID-19 pandemic, which reduced the number of biopsies performed and could have contributed to the differences observed between prophylaxis groups.

## CONCLUSIONS

Letermovir showed substantial advantages over valganciclovir in adherence, safety, and tolerability in kidney transplant recipients aged 65 y or older who were CMV D^+^/R^−^. The advantages of letermovir should be considered for the management of this older patient population in need of optimized care.

## ACKNOWLEDGMENTS

Medical writing and editorial assistance were provided under the direction of the authors by Seth Hurley, PhD, and Jenna Lewis, MA, ELS, Fingerpaint Medical. This assistance was funded by Merck Sharp & Dohme LLC, a subsidiary of Merck & Co Inc, Rahway, NJ.

## Supplementary Material


