# Supplementary material for: Favorable Adherence and Tolerability of Letermovir Versus Valganciclovir for Cytomegalovirus Prophylaxis in Kidney Transplant Recipients Aged 65 y or Older: Findings From a Randomized, Phase 3 Trial
Source: Transplant Direct. 2026 Aug 21;12(9):e1990. doi: 10.1097/TXD.0000000000001990 (PMC13502855; doi:10.1097/TXD.0000000000001990)

Favorable Adherence and Tolerability of Letermovir Versus Valganciclovir for Cytomegalovirus Prophylaxis in Recipients of Kidney Transplants Aged 65 Years or Older: Findings From a Randomized, Phase 3 Trial

Suphamai Bunnapradist, MD[1]; Flavio Vincenti, MD[2]; Oliver Witzke, Univ-Prof Dr med[3]; Stefan Schneeberger, MD[4]; M. Hong Nguyen, MD[5]; Natalya Broyde, MS[6]; Christopher L. Gilbert, BS[6]; Barbara Haber, MD[6]

[1]Division of Kidney Transplantation, David Geffen School of Medicine, University of California, Los Angeles, CA, USA; [2]Division of Nephrology, University of California, San Francisco School of Medicine, San Francisco, CA, USA; [3]Department of Infectious Diseases and Nephrology, West German Centre of Infectious Diseases and Immunology, University Medicine Essen, University Duisburg-Essen, Essen, Germany; [4]Department of General, Transplant and Thoracic Surgery, Medical University of Innsbruck, Innsbruck, Austria; [5]Department of Medicine, University of Pittsburgh, Pittsburgh, PA, USA; [6]Merck & Co., Inc., Rahway, NJ, USA

SUPPLEMENTARY APPENDIX

Contents

SUPPLEMENTARY METHODS 3
SUPPLEMENTARY TABLES 4
Table S1. 4
Table S2. 5
Table S3. 6
Table S4. 7
Table S5. 8
Table S6. 10
SUPPLEMENTARY FIGURES 11
Figure S1. 11
Figure S2. 12
Figure S3. 13
Figure S4. 14
Figure S5. 15
Figure S6. 16
Figure S7. 17

SUPPLEMENTARY METHODS

Investigators were strictly instructed to dose valganciclovir and acyclovir (and their respective matching placebos) based on creatinine clearance (CrCl) as indicated in the prescribing information and treatment guidelines.[1-4] Eligible participants were required to have a CrCl >10 mL/min (calculated with Cockcroft-Gault equation). Individuals receiving dialysis at randomization were excluded. Study treatment (including all agents) could be temporarily interrupted if postrandomization CrCl decreased to ≤10 mL/min or if dialysis was required for up to 7 consecutive days. Treatment could be resumed once CrCl exceeded 10 mL/min and dialysis was no longer necessary, provided that no more than 7 consecutive days of prophylaxis were missed.

References: 1. Valcyte [package insert]. San Francisco, CA, USA: Genetech USA, Inc; 2021. **2.** Zovirax [package insert]. Research Triangle Park, NC, USA: GlaxoSmithKline; 2005. **3.** Gold D, Corey L. Acyclovir prophylaxis for herpes simplex virus infection. *Antimicrob Agents Chemother.* 1987;31(3):361-367. **4.** Kotton CN, Kumar D, Manuel O, et al. The fourth international consensus guidelines on the management of cytomegalovirus in solid organ transplantation. *Transplantation.* 2025;109(7):1066-1110.

SUPPLEMENTARY TABLES

Table S1. Demographic and Baseline Characteristics for Participants Aged ≥65 Years (APaT)

Parameter	LET n=48	VGCV n=55
Sex, n (%)		
Male	32 (67)	33 (60)
Female	16 (33)	22 (40)
Age, mean (SD), y	69.7 (3.9)	69.7 (3.9)
≥75 y, n (%)	6 (12)	8 (15)
Race, n (%)		
Black or African American	0	1 (2)
White	48 (100)	54 (98)
Weight, mean (SD), kg	81.3 (16.9)	80.9 (15.1)
Donor type, n (%)		
Living related	3 (6)	4 (7)
Living not related	10 (21)	5 (9)
Deceased	35 (73)	46 (84)
Ex vivo time, mean (SD), h[a]	3.1 (1.3)	2.7 (0.9)
Use of lymphocyte-depleting induction immunosuppression, n (%)[b]		
Use	18 (38)	26 (47)
Antithymocyte globulin use[c]	17 (94)	23 (88)
No use	30 (62)	29 (53)
Primary reason for transplant, n (%)		
Diabetes or diabetic nephropathy	12 (25)	15 (27)
Hypertension	6 (12)	12 (22)
Congenital cystic kidney disease	8 (17)	7 (13)
Glomerulonephritis	3 (6)	5 (9)
Chronic kidney disease or ESRD	4 (8)	2 (4)
Immunoglobulin A nephropathy	2 (4)	5 (9)
Urinary obstruction	2 (4)	0
Renal atrophy	1 (2)	0
Tubulointerstitial nephritis	1 (2)	0
Alport syndrome	0	1 (2)
Other	9 (19)	8 (15)

APaT, all participants as treated; ESRD, end-stage renal disease; LET, letermovir; VGCV, valganciclovir. [a]n=34 and n=45 participants with data in the LET and VGCV groups, respectively. [b]Horse- or rabbit-derived antithymocyte globulin or alemtuzumab. Stratum based on confirmation of highly cytolytic antilymphocyte immunotherapy received at time of transplantation. [c]Percentage calculated using the number of participants receiving lymphocyte-depleting induction immunosuppression as the denominator.

Table S2. Key Demographic and Baseline Characteristics for Participants Aged ≥65 and <65 Years (APaT)

	Aged ≥65 years		Aged <65 years	
**Parameter**	**LET n=48**	**VGCV n=55**	**LET n=244**	**VGCV n=242**
Sex, n (%)				
Male	32 (67)	33 (60)	181 (74)	176 (73)
Female	16 (33)	22 (40)	63 (26)	66 (27)
Age, mean (SD), y	69.7 (3.9)	69.7 (3.9)	45.6 (12.6)	45.1 (12.8)
Race, n (%)				
American Indian or Alaska Native	0	0	3 (1)	4 (2)
Asian	0	0	4 (2)	10 (4)
Black or African American	0	1 (2)	21 (9)	32 (13)
White	48 (100)	54 (98)	205 (84)	189 (78)
Multiple races	0	0	9 (4)	6 (2)
Missing	0	0	2 (1)	1 (<1)
Weight, mean (SD), kg	81.3 (16.9)	80.9 (15.1)	83.0 (19.5)	83.1 (21.1)
Donor type, n (%)				
Living related	3 (6)	4 (7)	53 (22)	60 (25)
Living not related	10 (21)	5 (9)	55 (23)	46 (19)
Deceased	35 (73)	46 (84)	136 (56)	136 (56)
Ex vivo time, mean (SD), h[a]	3.1 (1.3)	2.7 (0.9)	2.9 (1.7)	3.0 (2.1)

APaT, all participants as treated; LET, letermovir; VGCV, valganciclovir. [a]n=34 and n=45 participants aged ≥65 years with data in the LET and VGCV groups, respectively; n=136 and n=133 participants aged <65 years with data in the LET and VGCV groups, respectively.

Table S3. Participants With Concomitant Immunosuppressant Medications During Prophylaxis (APaT)

	No. of participants (%)	
**Medication**	**LET n=48**	**VGCV n=55**
Tacrolimus	44 (92)	51 (93)
Mycophenolate mofetil	35 (73)	37 (67)
Prednisone	31 (65)	32 (58)
Mycophenolate sodium	7 (15)	21 (38)
Prednisolone	12 (25)	13 (24)
Methylprednisolone	11 (23)	11 (20)
Basiliximab	13 (27)	9 (16)
Tacrolimus monohydrate	10 (21)	8 (15)
Mycophenolic acid	7 (15)	8 (15)
Antithymocyte immunoglobulin	6 (12)	1 (2)
Everolimus	5 (10)	7 (13)
Methylprednisolone sodium succinate	3 (6)	4 (7)
Ciclosporin	1 (2)	3 (5)
Azathioprine	1 (2)	2 (4)
Antithymocyte immunoglobulin (rabbit)	1 (2)	1 (2)
Sirolimus	0	4 (7)
Dexamethasone	0	1 (2)
Meprednisone	0	1 (2)

APaT, all participants as treated; LET, letermovir; VGCV, valganciclovir.

Table S4. Incidence of Neutropenia or Leukopenia Stratified by Use vs Non-Use of Lymphocyte-Depleting Induction Therapy (APaT)

	No. of participants (%)	
**Parameter**	**LET n=48**	**VGCV n=55**
Participants with one or more leukopenia or neutropenia events	15/48 (31)	34/55 (62)
Use of lymphocyte-depleting induction immunosuppression[a]	7/18 (39)	18/26 (69)
Use of antithymocyte globulin[a]	6/17 (35)	16/23 (70)
No use of lymphocyte-depleting induction immunosuppression[a]	8/30 (27)	16/29 (55)

APaT, all participants as treated; LET, letermovir; VGCV, valganciclovir. [a]Percentages are expressed as the number of participants with one or more leukopenia or neutropenia events (numerator) over the number with or without lymphocyte-depleting induction immunosuppression use (denominator; presented in Table S1).

Table S5. Rehospitalizations Due to SAEs Through Week 52 (APaT)[a]

	No. of participants (%)	
**Cause**	**LET n=24**	**VGCV n=38**
Blood and lymphatic system disorders	1 (4)	2 (5)
Febrile neutropenia	0	1 (3)
Hypoplastic anemia	1 (4)	0
Neutropenia	0	1 (3)
Cardiac disorders	2 (8)	3 (8)
Atrial fibrillation	1 (4)	1 (3)
Atrial flutter	1 (4)	0
Cardiac failure congestive	0	1 (3)
Supraventricular tachycardia	0	1 (3)
Gastrointestinal disorders	3 (12)	2 (5)
Diarrhea	0	1 (3)
Fecaloma	1 (4)	0
Esophagitis	1 (4)	0
Retroperitoneal hematoma	1 (4)	0
Vomiting	0	1 (3)
General disorders and administration site conditions	3 (12)	0
Discomfort	1 (4)	0
General physical health deterioration	1 (4)	0
Pyrexia	1 (4)	0
Immune system disorders	3 (12)	0
Kidney transplant rejection	2 (8)	0
Transplant rejection	1 (4)	0
Infections and infestations	2 (8)	6 (16)
COVID-19 pneumonia	1 (4)	0
Clostridium difficile infection	0	1 (3)
Cytomegalovirus gastritis	1 (4)	0
Cytomegalovirus infection	0	1 (3)
Infected urinoma	0	1 (3)
Influenza	0	1 (3)
Respiratory tract infection	0	1 (3)
Urosepsis	0	2 (5)
Injury, poisoning, and procedural complications	1 (4)	4 (11)
Complications of transplanted kidney	1 (4)	0
Humerus fracture	0	1 (3)
Incision site pain	0	1 (3)
Renal transplant failure	0	1 (3)
Wound dehiscence	0	1 (3)

Investigations	0	1 (3)
White blood cell count decreased	0	1 (3)
Metabolism and nutritional disorders	1 (4)	2 (5)
Decreased appetite	0	1 (3)
Diabetes mellitus	1 (4)	0
Hypocalcemia	0	1 (3)
Nervous system disorders	1 (4)	1 (3)
Amnesia	0	1 (3)
Coma	1 (4)	0
Tremor	0	1 (3)
Renal and urinary disorders	3 (12)	5 (13)
Acute kidney injury	0	4 (11)
End-stage renal disease	0	1 (3)
Subcapsular renal hematoma	1 (4)	0
Urinary incontinence	1 (4)	0
Urinoma	1 (4)	0
Reproductive system and breast disorders	1 (4)	0
Vulvovaginal pruritus	1 (4)	0
Respiratory, thoracic, and mediastinal disorders	1 (4)	1 (3)
Acute pulmonary edema	1 (4)	1 (3)
Vascular disorders	2 (8)	3 (8)
Deep vein thrombosis	1 (4)	0
Hypertensive crisis	0	1 (3)
Lymphocele	1 (4)	2 (5)

APaT, all participants as treated; LET, letermovir; SAE, serious adverse event; VGCV, valganciclovir. [a]Among participants experiencing rehospitalizations.

Table S6. Allograft Dysfunction and/or Rejection Through Weeks 28 and 52 Posttransplant (FAS)

	Percentage of participants (95% CI)	
**Participants with allograft dysfunction and/or rejection**	**LET n=47**	**VGCV n=55**
Any allograft dysfunction and/or rejection		
Through Week 28 posttransplant	74 (60 to 86)	73 (59 to 84)
Through Week 52 posttransplant	77 (62 to 88)	73 (59 to 84)
Graft loss		
Through Week 28 posttransplant	0 (0 to 8)	0 (0 to 6)
Through Week 52 posttransplant	0 (0 to 8)	2 (0 to 10)
≥20% decline in posttransplant eGFR from Week 4 through Week 28 posttransplant[a]	17 (8 to 31)	18 (9 to 31)
eGFR <60 at Week 28 posttransplant	70 (55 to 83)	71 (57 to 82)
Biopsy-proven acute renal graft rejection[b]		
Through Week 28 posttransplant	11 (4 to 23)	4 (0 to 12)
Through Week 52 posttransplant	15 (6 to 28)	4 (0 to 12)

eGFR, estimated glomerular filtration rate; FAS, full analysis set; LET, letermovir; VGCV, valganciclovir. [a]eGFR was calculated using the 4-variable Modification of Diet In Renal Disease equation. The measurement closest to 4 weeks in the window from 2 to 6 weeks posttransplant was used for the 4 weeks baseline. [b]Performance of biopsy procedures was affected by the COVID-19 pandemic.

SUPPLEMENTARY FIGURES

Figure S1. P002 study design with key endpoints for the subpopulation aged ≥65 years. Doses of VGCV (or intravenous ganciclovir) and ACV were modified based on CrCl. ACV, acyclovir; BID, twice daily; CrCl, creatinine clearance; CMV, cytomegalovirus; LET, letermovir; QD, once daily; VGCV, valganciclovir. [a]Given orally or intravenously. [b]If participant was receiving concomitant cyclosporine A, LET 240 mg QD was administered (orally or intravenously). [c]ACV 400 mg (orally) or 250 mg/m^2 (intravenously). [d]Given orally; alternatively, ganciclovir 5 mg/kg QD (intravenously) could be administered.

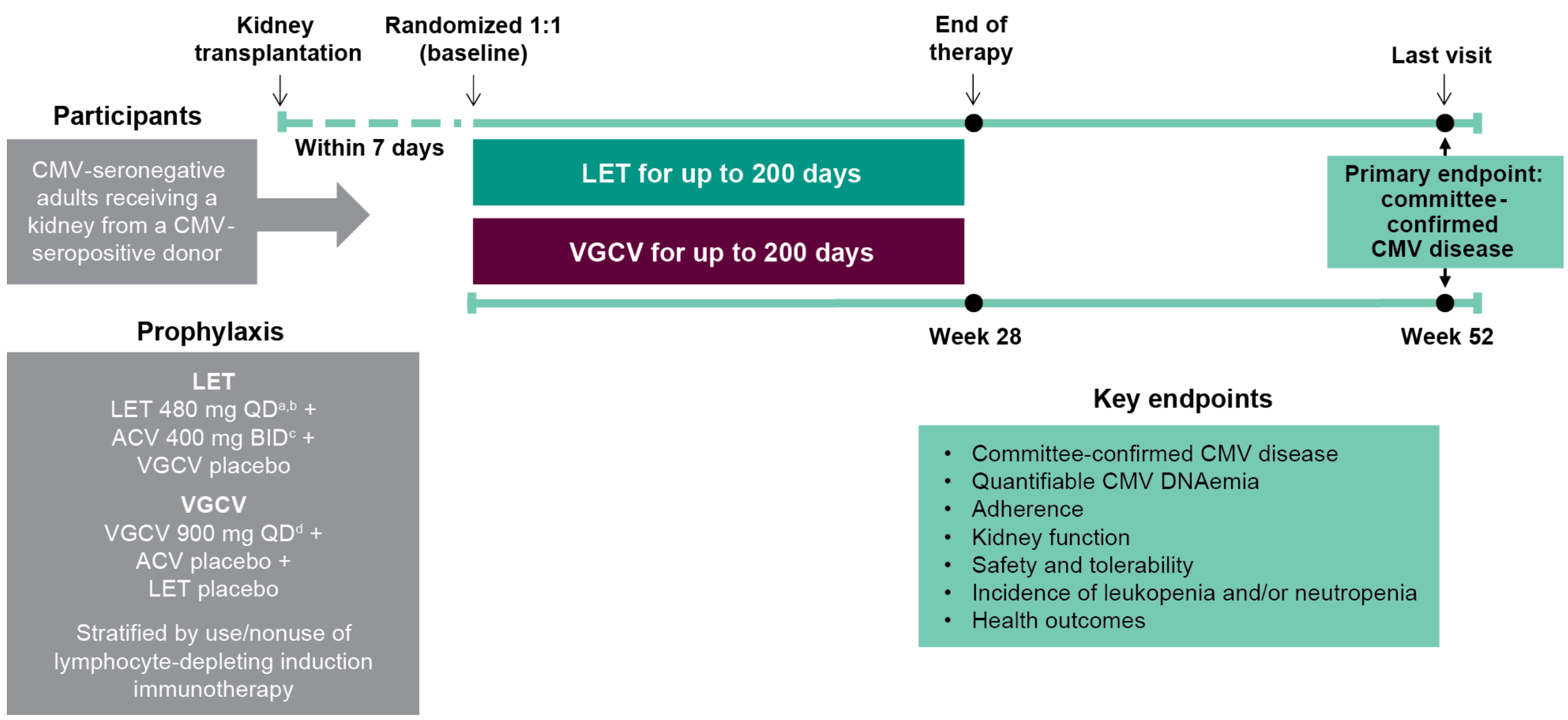

Figure S2. Disposition among participants aged ≥65 years through Week 52 posttransplant. APaT, all participants as treated; CMV, cytomegalovirus; FAS, full analysis set; LET, letermovir; VGCV, valganciclovir. [a]The overall study disposition has been published previously (Limaye et al. *JAMA.* 2023;330:33-42).

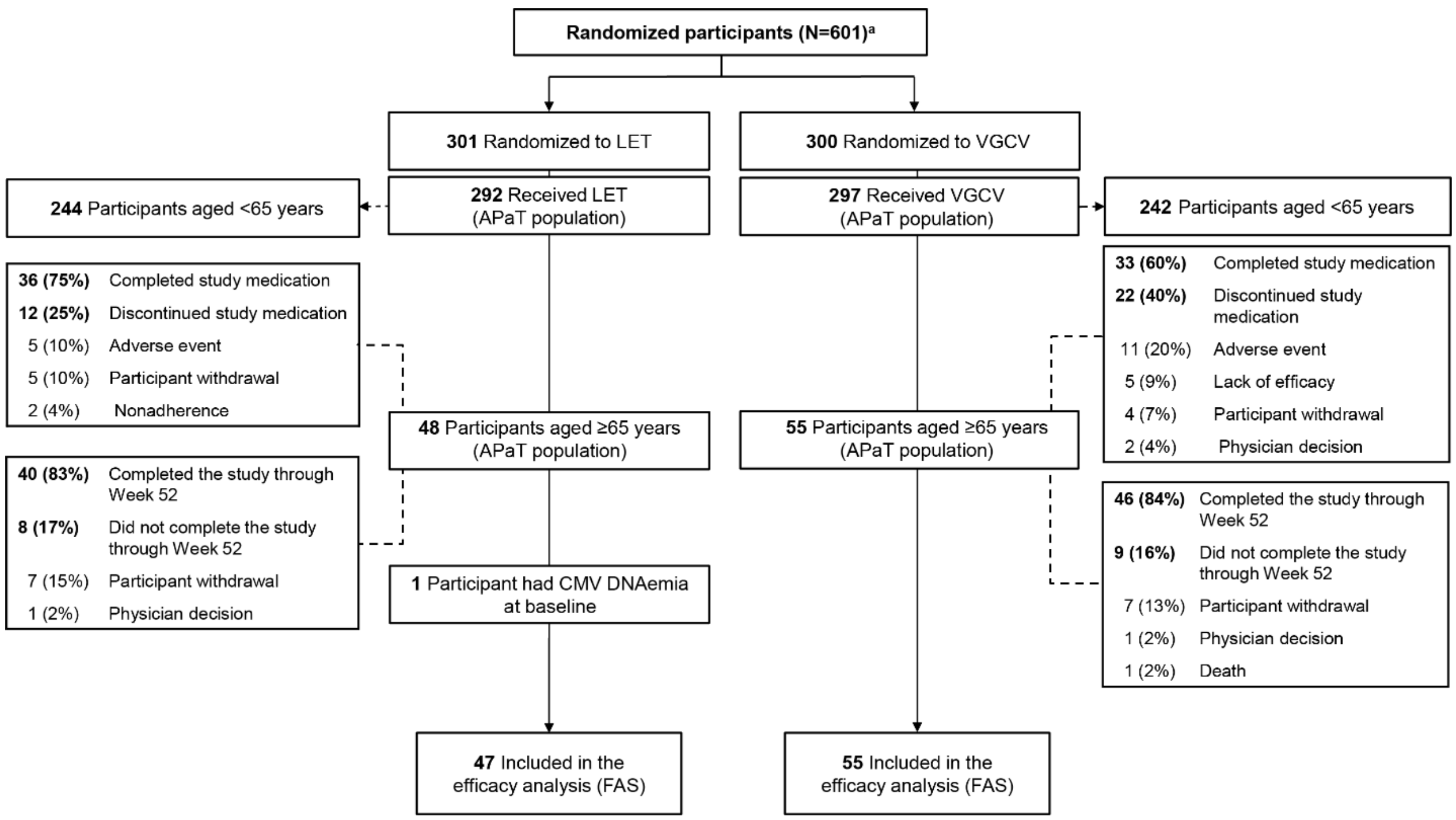

Figure S3. Percentage of participants with quantifiable CMV DNAemia through Weeks 28 and 52 posttransplant (FAS). CMV, cytomegalovirus; FAS, full analysis set; LET, letermovir; VGCV, valganciclovir. [a]Quantifiable CMV DNAemia was defined as any detected CMV with a numeric value (≥137 IU/mL).

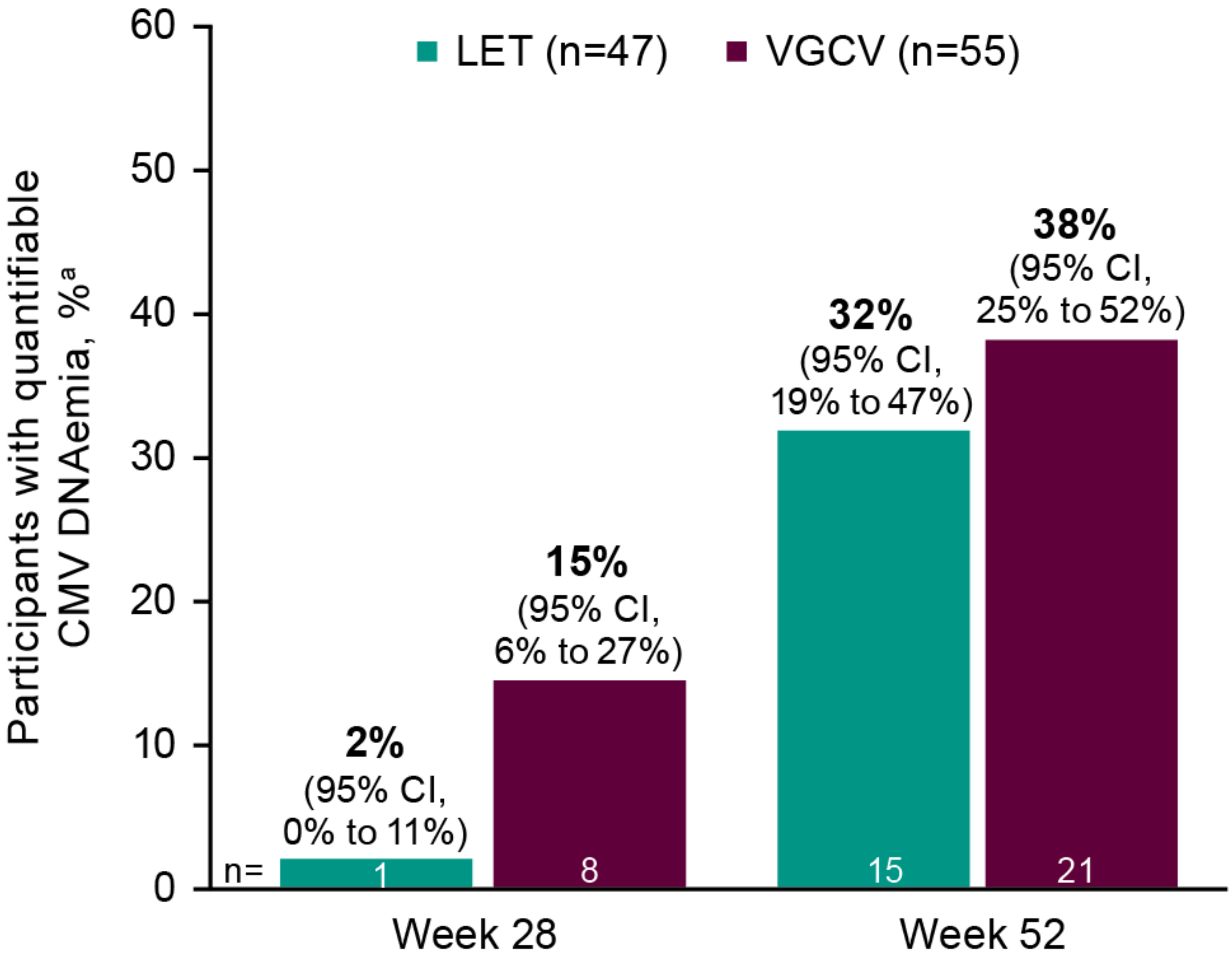

Figure S4. Dose frequency and adherence through Week 28 posttransplant (APaT). The percentage of participants prescribed once-daily or intermittent LET or VGCV dosing, along with dosing of matching placebos, are shown in the bar graph. The table below the bar graph shows adherence rates to LET or VGCV (the active CMV antivirals). Investigator-prescribed dose modifications based on CrCl were not counted as nonadherent days. APaT, all participants as treated; CMV, cytomegalovirus; CrCl, creatinine clearance; LET, letermovir; VGCV, valganciclovir. [a]Received LET or LET placebo dose once daily. [b]Received VGCV 450 or 900 mg or VGCV placebo dose once daily. [c]Received intermittent VGCV (450 mg every 2 days or twice weekly) during all or part of prophylaxis.

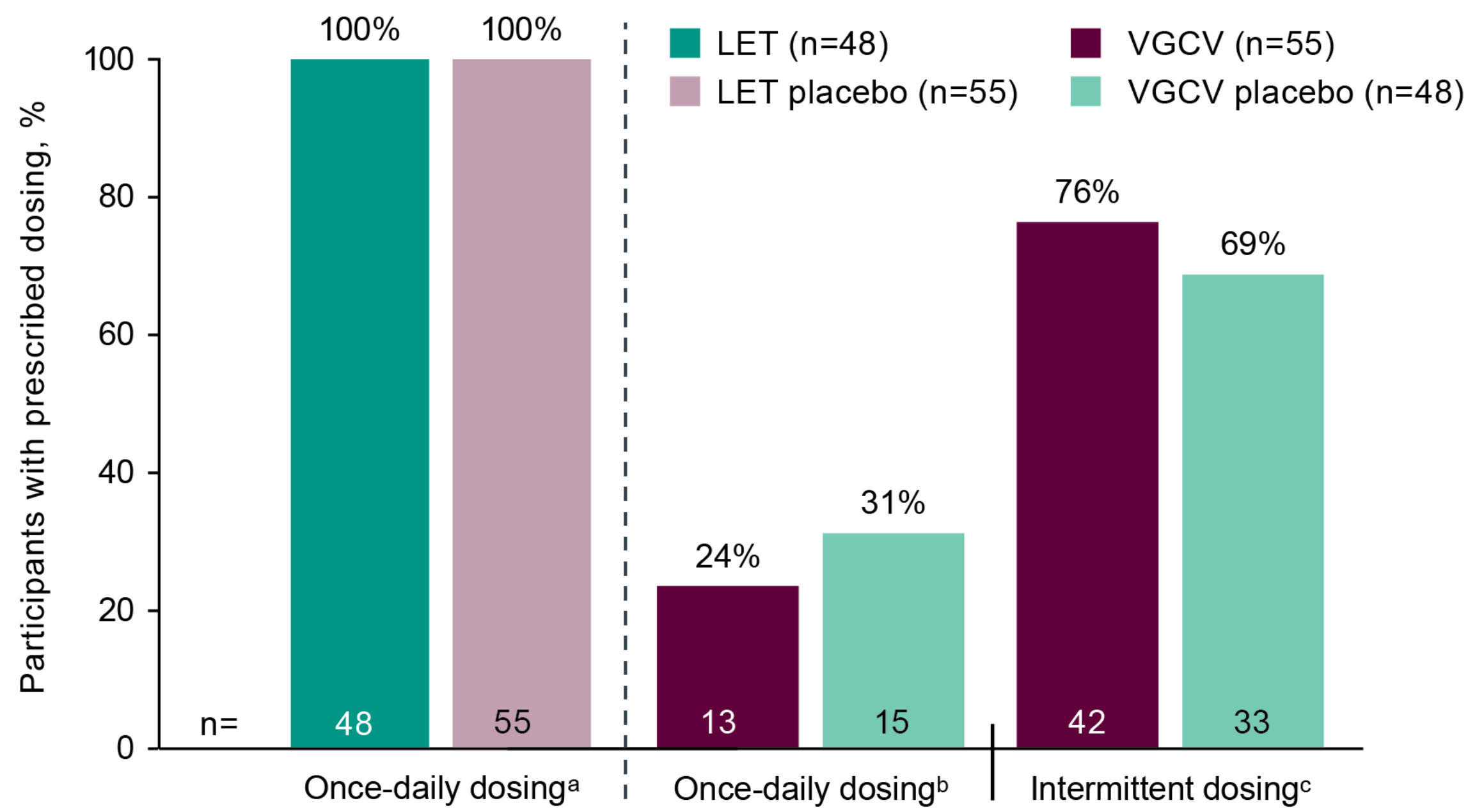

Median adherence to LET or VGCV, %	100		100		82	
Participants with 100% adherence, n (%)	26 (54)		4 (31)		0	
Participants with 90% to <100% adherence, n (%)	20 (42)		8 (62)		15 (36)	
Participants with 75% to <90% adherence, n (%)	2 (4)		0		11 (26)	
Participants with <75% adherence, n (%)	0		1 (8)		16 (38)	

Figure S5. Creatinine clearance in participants aged <65 and ≥65 years through Week 28 posttransplant for the **(A)** LET and **(B)** VGCV groups (APaT). APaT, all participants as treated; CrCl, creatinine clearance; LET, letermovir; VGCV, valganciclovir.

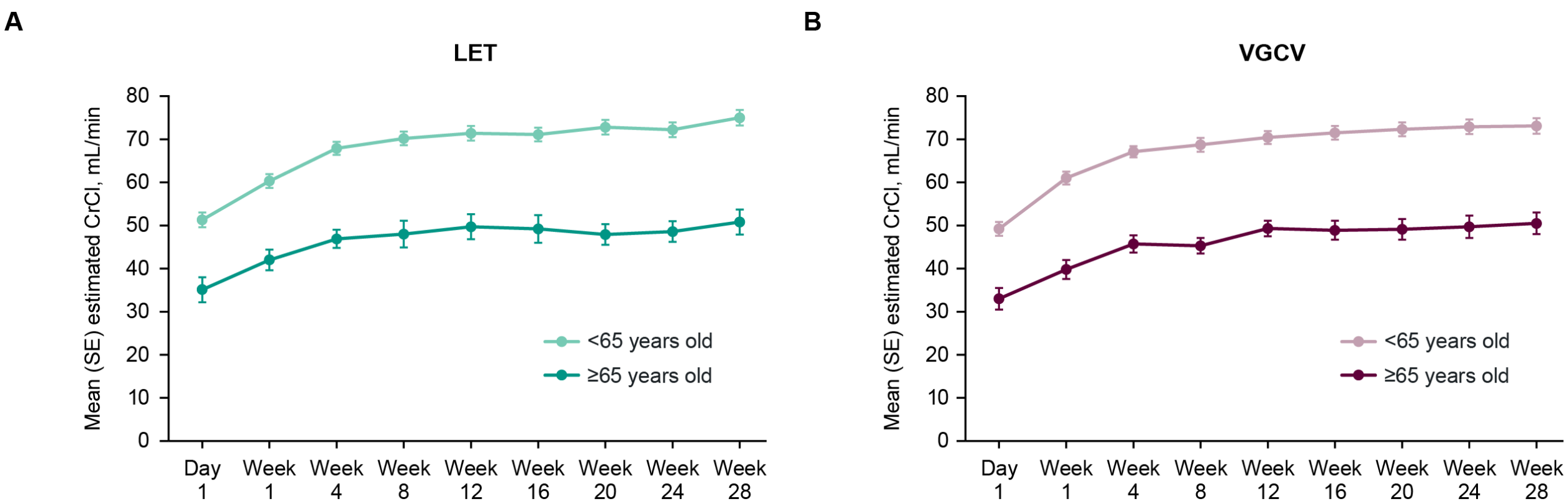

<65 years old: n=	240	226	216	215	215	212	209	206	198
≥65 years old: n=	48	45	41	37	34	35	34	37	35

<65 years old: n=	234	221	227	206	217	215	214	204	202
≥65 years old: n=	52	52	52	49	48	48	45	46	44

Figure S6. Estimated glomerular filtration rate in participants aged <65 and ≥65 years through Week 28 posttransplant for the **(A)** LET and **(B)** VGCV groups (APaT). APaT, all participants as treated; eGFR, estimated glomerular filtration rate; LET, letermovir; VGCV, valganciclovir.

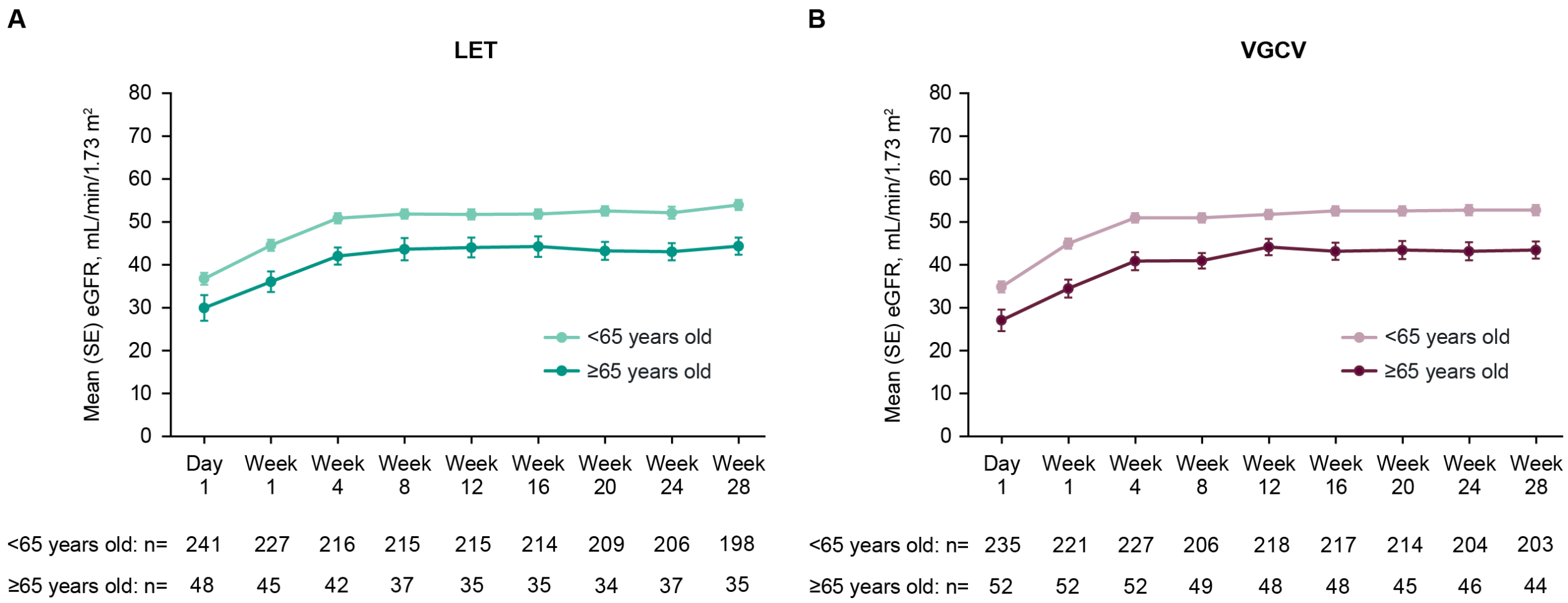

Figure S7. Rehospitalizations through Week 52 posttransplant by any rehospitalization, number of rehospitalizations, and rehospitalization due to CMV disease (FAS). CMV, cytomegalovirus; FAS, full analysis set; LET, letermovir; VGCV, valganciclovir. [a]One of 4 participants had >1 rehospitalization. [b]One of 10 participants had >1 rehospitalization.

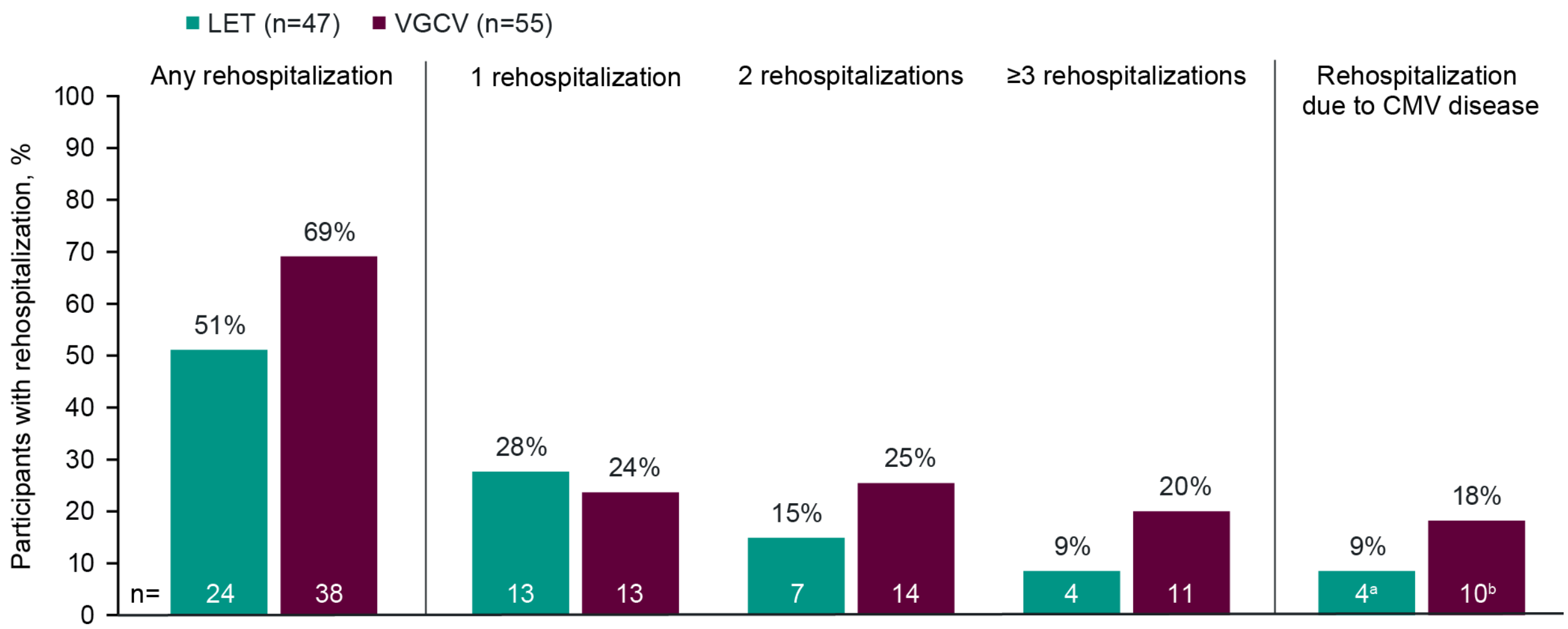

Supplement: Supplementary file 1 [file txd-12-e1990-s001.pdf]
